# Sexually dimorphic neuronal inputs to the neuroendocrine dopaminergic system governing prolactin release

**DOI:** 10.1111/jne.12781

**Published:** 2019-09-02

**Authors:** Francisco F. Esteves, Diogo Matias, Ana R. Mendes, Bertrand Lacoste, Susana Q. Lima

**Affiliations:** ^1^ Champalimaud Research Programa Champalimaud de Neurociências Lisboa Portugal

**Keywords:** arcuate nucleus, connectomics, dopamine, monosynaptic retrograde tracing, prolactin, sexually dimorphic

## Abstract

Prolactin (PRL) is a pleiotropic hormone that was identified in the context of maternal care and its release from the anterior pituitary is primarily controlled by neuroendocrine dopaminergic (NEDA) neurones of the arcuate nucleus of the hypothalamus. The sexually dimorphic nature of PRL physiology and associated behaviours is evident in mammals, even though the number and density of NEDA neurones is reported as not being sexually dimorphic in rats. However, the underlying circuits controlling NEDA neuronal activity and subsequent PRL release are largely uncharacterised. Thus, we mapped whole‐brain monosynaptic NEDA inputs in male and female mice. Accordingly, we employed a rabies virus based monosynaptic tracing system capable of retrogradely mapping inputs into genetically defined neuronal populations. To gain genetic access to NEDA neurones, we used the dopamine transporter promoter. Here, we unravel 59 brain regions that synapse onto NEDA neurones and reveal that male and female mice, despite monomorphic distribution of NEDA neurones in the arcuate nucleus of the hypothalamus, receive sexually dimorphic amount of inputs from the anterior hypothalamic nucleus, anteroventral periventricular nucleus, medial preoptic nucleus, paraventricular hypothalamic nucleus, posterior periventricular nucleus, supraoptic nucleus, suprachiasmatic nucleus, lateral supramammillary nucleus, tuberal nucleus and periaqueductal grey. Beyond highlighting the importance of considering sex as a biological variable when evaluating connectivity in the brain, these results illustrate a case where a neuronal population with similar anatomical distribution has a subjacent sexually dimorphic connectivity pattern, potentially capable of contributing to the sexually dimorphic nature of PRL release and function.

## 1 INTRODUCTION

Mammals show sexual dimorphism in social and sexual behaviours. Despite the relevance of sex‐specific behaviours for the reproductive success of each species, the brain circuitry underlying sex‐specific behaviours is largely unknown. Sexual dimorphism of the mammalian brain arises from genetic differences at the level of sex chromosomes. These differences result in the maturation of sexually dimorphic neural circuits as a result of the differential organisational action of gonadal sex hormones.^1^, ^2^, ^3^ Nonetheless, sex as a biological variable remains underexplored in neural circuit mapping studies.^4^

Prolactin (PRL) is a non‐gonadal pleiotropic peptide hormone primarily released by the anterior pituitary gland.^5^ Originally named after its role in lactation,^6^ PRL is released in response to innumerous external factors and physiological states.^7^ Although some, such as stress,^8^, ^9^ are shared by both sexes, others are sexually dimorphic. The latter ones include the release of PRL in response to nipple stimulation in lactating females,^5^ the PRL circadian surges in naturally cycling females,^10^, ^11^ and PRL release during copulation in males.^12^ In females, PRL is fundamental in organising a series of physiological and behavioural programmes that prepare individuals for motherhood: it decreases female receptivity after fertilisation,^13^ and also promotes food intake^14^ and maternal behaviour.^15^, ^16^ All of these programmes are fundamental to ensure the survival of the progeny. By contrast, the role of PRL in male‐specific behaviours is less well understood, although it has been proposed that PRL release during copulation regulates libido.^17^

PRL is primarily produced and released into the bloodstream by specialised cells of the anterior pituitary, the lactotrophs.^18^ Its receptor, the prolactin receptor (PRLr), can signal through a multitude of second messenger cascades when activated.^19^ The PRLr has widespread expression in both the male and female mouse brain.^20^, ^21^ Although PRLr expression is concentrated in the rostral and mediobasal hypothalamus, extra‐hypothalamic responses to PRL can also be detected in the medial amygdala, bed nucleus of the stria terminalis, lateral septum and others.^19^, ^21^ Some of these extra‐hypothalamic regions are known to be important for the modulation of sex‐specific behaviours.^22^

Several inhibitory and stimulatory factors control the release of PRL,^22^ although the most important site of regulation resides in the neuroendocrine dopaminergic neurones (NEDA) of the medial basal hypothalamus, the majority of which are located in the arcuate nucleus of the hypothalamus (ARH).^18^ NEDA neurones inhibit the production and release of PRL by lactotrophs via dopamine transmission into the blood in response to PRL itself. Suppression of dopamine discharge by NEDA neurones leads to disinhibition of lactotrophs, which quickly release PRL into circulation, thus establishing a self‐inhibitory feedback loop.^7^, ^18^, ^23^

Several hypotheses exist regarding the regulation of NEDA neural activity based on the behavioural and physiological conditions that lead to PRL release and on the neurochemicals that NEDA neurones respond to in *ex vivo* brain slices.^7^, ^24^ Nevertheless, the sources of non‐local brain‐derived signals are yet to be uncovered. A notable exception is the suprachiasmatic nucleus, whose neurones synapse onto NEDA^25^ neurones and influence their circadian activity in female rats.^26^ Other brain areas outside the ARH modulating the activity of NEDA neurones are mostly unknown and, importantly, their role in male PRL physiology remains a mystery.

NEDA neurones express the *Th* (tyrosine hydroxylase) and *slc6A3* (dopamine transporter – *dat*) genes and correspond to the A12 and A14 groups of Dahlström and Fuxe.^27^ Different types of NEDA neurones have neurohemal synapses in fenestrated capillary beds of either the external layer of the median eminence (ME) (tuberoinfundibular dopaminergic neurones), the intermediate lobe of the pituitary (periventricular hypophyseal dopaminergic neurones) or the neurohypophysis (tuberhypophyseal dopaminergic neurones).^28^ Besides dopamine, NEDA neurones produce and release several other neurotransmitters and modulators, suggesting an even broader biological role for these hypothalamic neurones.^29^, ^30^

Given the role of NEDA in prolactin‐related sex‐specific physiology and behaviours, we set out to characterise: (i) the number and distribution of NEDA neurones in the dorso‐medial arcuate nucleus of female and male mice and (ii) the brain regions contributing monosynaptic inputs to NEDA neurones in male and female mice. To genetically access NEDA neurones, we took advantage of the DAT promoter to first label and quantify NEDA neurones in both sexes. Secondly, using the rabies virus (RV) monosynaptic tracing system,^31^, ^32^ we performed a whole‐brain survey of regions that harbor neurones directly synapsing onto NEDA neurones. The RV monosynaptic tracing system consists of a recombinant RV and two different adeno‐associated viruses (AAV) that are delivered into a transgenic mouse. The RV is modified in three important ways: (i) to confine the RV spread to a single synaptic jump, the RV envelope glycoprotein (G‐protein) necessary for the virus’ transsynaptic spread is deleted and complemented in *trans* by way of a first helper AAV that expresses the G‐protein in a Cre‐dependent manner via the FLEx switch (ie, the G‐protein gene is flanked by inverted *lox*P sites). The Cre recombinase is expressed from the genome of an appropriate transgenic mouse line. This modification renders the G‐deleted RV capable of jumping transsynaptically exclusively from cells that were co‐infected with the G‐protein coding AAV. The RV does not spread further than the first‐order monosynaptic partners because these cells do not contain the G‐protein necessary to assemble functional RV particles; (ii) to make the RV transfection cell type‐specific, an envelope protein from an avian sarcoma leucosis virus (EnvA) was introduced in the RV genome. Because EnvA is not recognised by mammalian receptors, the RV can only enter mammalian cells previously transfected by a second helper Cre‐dependent AAV that provides the EnvA receptor TVA. Cell specificity of RV infection is thus achieved because only cells expressing Cre recombinase and previously transfected by AAVs expressing TVA can be infected by the RV; (iii) the final crucial modification is the introduction of a reporter gene (eGFP [enhanced green fluorescent protein]) into the RV genome to allow visualisation of RV spread. Therefore, to deliver the RV monosynaptic tracing system, first a stereotactic injection of two different AAVs in the brain of a Cre recombinase expressing transgenic mouse is performed. Then, after allowing sufficient time for viral protein Cre‐dependent expression, a second round of stereotactic injections is performed on the animal in the same brain region to deliver the RV (Figure 2A).

By employing the RV monosynaptic tracing system, we reveal that, despite monomorphic distribution of NEDA neurones, there is sexual dimorphism in the monosynaptic inputs to these neurones. In particular, we show that the anterior hypothalamic nucleus (AHN), aqnteroventral periventricular nucleus (AVPV), medial proptic nucleus (MPN), paraventricular hypothalamic nucleus (PVH), paraventricular hypothalamic nucleus preoptic (PVpo), supraoptic nucleus (SO), suprachiasmatic nucleus (SCH), supramammillary nucleus lateral part (SUMl), tuberal nucleus (TU) and periaqueductal grey (PAG) (for abbreviations, see Figure 3C) are sexually dimorphic in the relative contribution of their projections to NEDA neurones, thus setting the stage for understanding the mechanisms underlying the regulation of PRL release and its function in both sexes.

## 2 MATERIALS AND METHODS

### 2.1 Animals

Animals were kept under a reversed 12:12 h dark/light cycle (lights on 20.00 h) with access to food and water available ad lib. in temperature‐controlled rooms (22‐24°C). The animals were group housed until the first surgical procedure and isolated thereafter.

All mice used were sexually naive adults aged between 3‐4 months.

AAV and RV injections were performed in 44 male and 34 female *dat‐cre* 33 (JAX stock #006660) heterozygous mice backcrossed and maintained in a C57BL/6 background. *dat‐cre* mice were also crossed with a tdTomato expressing line^34^ (JAX stock #007905) and 10 double heterozygous animals were used (n_females_ = 5, n_males_ = 5). Wild‐type littermates were used for control and immunohistochemistry experiments.

All procedures were reviewed and performed in accordance with the Champalimaud Welfare Body and the Champalimaud Foundation Ethics Committee guidelines, and were also approved by the Portuguese National Authority for Animal Health.

### 2.2 Viral vectors

Viral vectors were purchased either from the Salk Institute Gene Transfer, Targeting and Therapeutics Core (La Jolla, CA, USA) (EnvA‐G deleted Rabies‐eGFP) or the University of North Carolina Vector Core (Chapel Hill, NC, USA) (AAV8‐CA‐FLEx‐RG and AAV8‐EF1a‐FLEx‐TVAmCherry). Viruses were injected at provided titer: 1.5‐1.77 × 10^8^ pfu mL for the RV; 3.3 × 10^12^ pfu mL for the AAV8‐CA‐FLEx‐RG; and 8 × 10^12^ pfu mL for AAV8‐EF1a‐FLEx‐TVAmCherry. For the AAV injections, AAV‐CAG‐FLEx‐RG and AAV‐EF1a‐FLEx‐TVAmCherry were mixed at a 1:1 ratio and injected together.

### 2.3 Stereotaxic virus injections and histology

Mice were anaesthetised with 3% isoflurane in oxygen and head fixed in a stereotaxic frame using ear‐bars (Kopf, Tujunga, CA, USA). During surgery, anaesthesia was maintained using 1.5% isoflurane. After a head skin incision, the cranium was exposed and a hole drilled for pipet insertion at appropriate coordinates. Pulled capillaries (length 3‐1/2 inches [9 cm]; inner diameter 0.53, outer diameter 1.14 mm; tip diameter 40 μm; Drummond Scientific, Broomall, PA, USA) were used to inject 195‐1000 nL of AAV mixture at ‐1.45 mm or ‐1.3 mm posterior and 0.2 mm or 0.25 mm lateral to bregma, at a depth of ‐5.9 mm from the brain surface at a rate of 0.46 nL s^‐1^. Analgesic (buprenorphine 0.1 mg kg^‐1^) was administered post‐surgery at all times. After waiting 2 weeks to allow for viral expression, an injection of 50‐2000 nL of RV was performed at the same coordinates using the previous craniotomy. Animals were maintained in isolation for 1 week to allow viral expression before being killed. On the day of death, the animals were deeply anaesthetised and perfused transcardially with 0.01 mol L^‐1^ phosphate buffer (PBS), followed by a cold 4% paraformaldehyde solution (PFA) in 0.01 mol L^‐1^ PBS. Brains were removed from the skulls and stored overnight at 4°C in 4% PFA solution. The following day, after washing in 0.01 mol L^‐1^ PBS, whole brains were cut into 50‐μm‐thick coronal sections using a vibratome (VT1000S; Leica Microsystems, Wetzlar, Germany). Brain sections were immediately mounted on poly‐lysine‐coated glass slides and covered with Mowiol (Sigma‐Aldrich) mounting medium and a glass coverslip.

### 2.4 Immunohistochemistry for mCherry

To enhance mCherry detection, the coverslips were removed from the slides by immersing in 0.01 mol L^‐1^ PBS + Triton‐X 0.3% for 48 hours at room temperature. To further remove mounting media, the slides were washed in 0.01 mol L^‐1^ PBS three times for 5 minutes. A permeabilisation step was included that consisted of a 60‐minute wash with 0.01 mol L^‐1^ PBS + Triton‐X 0.4%. The primary antibody (mouse anti‐mCherry; AB167453; Abcam, Cambridge, MA, USA) was incubated at 1:200 in 0.01 mol L^‐1^ PBS + Triton‐X 0.4% and left for 96 h at 4°C in a wet chamber. To wash off the primary antibody, five washes of 5 minutes each in 0.01 mol L^‐1^ PBS were performed. Secondary antibody (Alexa Fluor rabbit anti‐mouse 647; ab150115; Abcam) was incubated in 0.01 mol L^‐1^ PBS + Triton‐X 0.4% at 1:1000 dilution for 3 hours at room temperature. Another set of five washes of 5 min each in 0.01 mol L^‐1^ PBS were performed to remove excess secondary antibodies. The slides were coverslipped with Mowiol mounting medium.

### 2.5 Imaging and data analysis

Brain sections were imaged using an automated slide scanner (AxioScan Z1; Carl Zeiss, Oberkochen, Germany). The locations and numbers of labelled neurones were manually determined using the Allen Brain Atlas as a reference (http://atlas.brain-map.org/). All procedures were performed using zen, version 2.0 (Carl Zeiss) and fiji (https://fiji.sc). To generate figures with representative images, brightness and contrast were adjusted using photoshop (Adobe Systems, San Jose, CA, USA) . Only animals targeted to the ARH and displaying a clear GFP signal were considered (see Results). Manual cell counts were stored as csv files and the data analysis was performed using custom Pyhton2.7 scripts (available upon request). Our analysis is focused on brain regions that showed GFP‐positive cells in at least three animals. The cell counts for each animal were normalised by dividing the number of neurones found in each region by the number of cells in the arcuate nucleus of the hypothalamus (ARH) (site of injection; see Results). The ARH was subsequently removed from analysis. Given our sample size (n_females_ = 5, n_males_ = 5), a single region sexual dimorphism is considered statistically significant at alpha = 0.05 when a Mann‐Whitney *U* test resulted in a critical *U* value equal to or smaller than 2 (two‐tailed test), and statistically significant at alpha = 0.01 when a Mann‐Whitney *U* test resulted in a critical *U* value equal to 0 (two‐tailed test).

## 3 RESULTS

### 3.1 Mouse arcuate nucleus NEDA populations are sexually monomorphic

**Figure 1:**
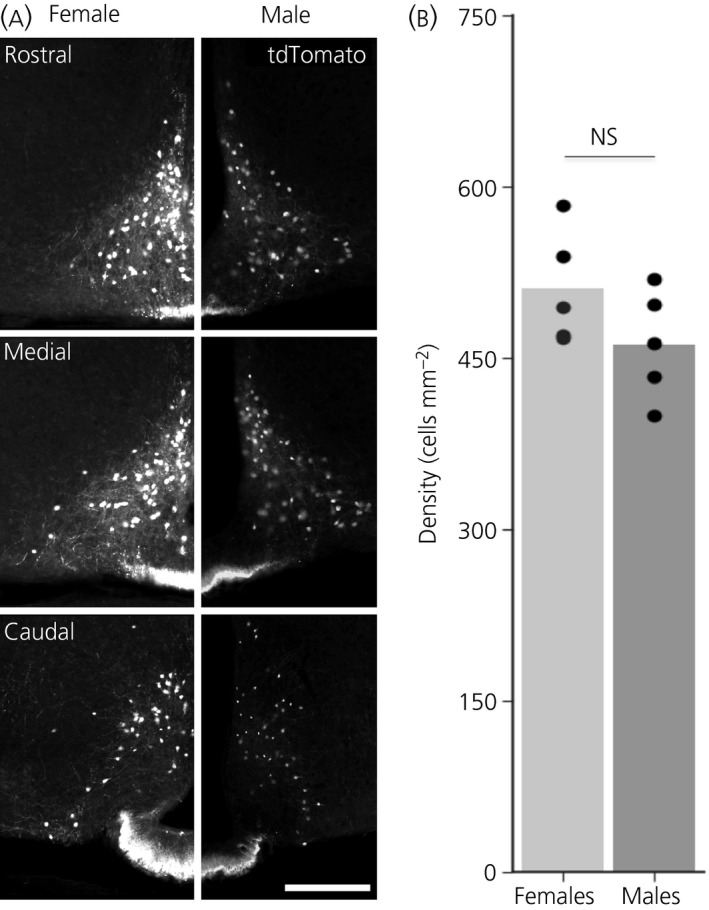
Male and female mice have similar distribution of neuroendocrine dopaminergic (NEDA) neurones. A, Representative images depicting tdTomato expressing cells in the arcuate nucleus of the hypothalamus (ARH) of female and male animals. Scale bar = 200 μm. B, Average density of tdTomato expressing cells observed in the dorsomedial ARH of female (n = 5) and male (n = 5) mice (NS, Mann‐Whitney *U* test not statistically significant)

The sexually dimorphic functions of PRL are exemplified by lactation associated with reproduction. In the vast majority of mammals, only the female sex displays this behaviour. This observation, together with the fact that female NEDA neurones release more dopamine into the circulation,^35^ raises the possibility that the observed differences might result from different numbers of NEDA neurones between the two sexes. To establish whether the NEDA population is sexually dimorphic regarding its distribution, we quantified the number of tdTomato‐positive cells in the ARH of both male and female mice expressing tdTomato under the control of the dopamine active transporter (dat*)* promoter (Figure 1A). The results obtained indicate that the number of dopamine active transporter (Dat)‐expressing NEDA neurones in the dorso‐medial ARH (dmARH) between males and females is not significantly different (Figure 1B), in agreement with what is reported for arcuate nucleus tyrosine hydroxylase‐positive neurones in rats,^36^ as well as a qualitative observation made in a previous study employing the same transgenic mouse line.^37^

### 3.2 Transsynaptic labelling of male and female NEDA monosynaptic inputs using RV

**Figure 2:**
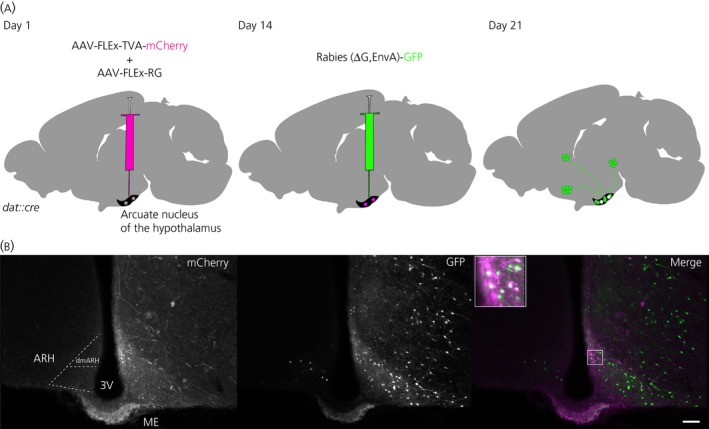
Identification of monosynaptic inputs to NEDA neuroendocrine dopaminergic (NEDA) neurones using rabies virus. A, Experimental design, mouse line and viral strains used. The arcuate nucleus of the hypothalamus (ARH) of male and female *dat::cre* mice were initially unilaterally injected with adeno‐associated virus (AAV) carrying the TVA and G transgene to restrict the starter population; 14 days later, animals were injected with the modified rabies virus. B, Representative image depicting starter cells (mCherry + green fluorescent protein [GFP], inset) in the dorsomedial ARH and rabies virus‐only positive cells (GFP). Scale bar = 100 μm

After establishing that female and male mice have similar numbers of NEDA neurones, we aimed to interrogate the pattern of monosynaptic inputs into this neuronal population in both sexes. Several brain regions have been implicated in the control of the activity of NEDA neurones in relationship to PRL metabolism and function.^23^ However, studies reporting regions with direct inputs into NEDA neurones are scarce.^25^, ^38^ To retrogradely label monosynaptic inputs to NEDA neurones, we employed a RV tracer system that allows mapping of monosynaptic inputs onto genetically defined neuronal populations (dopamine transporter expressing neurones, Dat neurones) of the ARH of male and female mice.^31^ This system employs two distinct helper Cre‐dependent AAVs that are injected first to limit the cell population infected by a subsequent injection of RV. One AAV carries an avian receptor transgene (TVA), the other AAV carries the rabies glycoprotein. Both viral transgenes require the presence of Cre recombinase to be expressed because inverted *lox*P sites flank them. AAVs infect all cells at the injection site, although only Cre‐positive cells produce both the TVA receptor required for transfection of a pseudotyped RV (RV‐ΔG, EnvA, eGFP) and the rabies glycoprotein required for RV synapse‐dependent spread. Because the RV will only infect the previously AAV infected cells, this system confines the transmission of retrogradely spreading RV to cells one synapse upstream of the initial Cre‐positive cells. Because both the AAV encoded TVA and the RV itself are tagged with mCherry and GFP respectively, the result is a Cre‐positive double‐labelled population, with the monosynaptic partners expressing GFP alone (Figure 2A, right). We injected AAVs unilaterally in the ARH of *dat‐cre* mice (Figure 2A, left) and, after waiting 2 weeks for expression of viral proteins, RV was injected in the same location (Figure 2A, middle). One week later after RV injection, the animals were killed and their brains processed for fluorescence detection. Thirty‐four animals did not show any fluorescence, most likely because one or both injections mistargeted and were confined to the closely juxtaposed third ventricle, instead of the ARH. In our analysis of the 45 animals displaying GFP signal, 21 showed very sparse labelling, 14 had sparse labelling in the ARH somas and ME axon terminals, and, instead, an extensive GFP signal in other nuclei, and 10 were selected for further processing (total cell counts are provided in the Supporting information, Table S1). These 10 animals were selected not only because the RV injection site was located near the ARH, but also because these brains displayed robust ARH soma and ME axon terminals signal as well as consistent GFP signal in several other brain regions. Additionally, of the eight animals for which mCherry antibody detection was carried out, TVA‐mCherry expression was found predominantly in NEDA containing regions (at least 50% starter cells in the ARH and periventricular hypothalamic nucleus). Besides mCherry‐GFP double‐positive cells (starter cells; example in Figure 2B, inset), the ARH contained many GFP‐only positive somas, which confirms the existence of extensive local inputs to NEDA neurones within the ARH.^23^ In this study, we focused our analysis on surveying the regions that presented GFP signal outside the ARH as a consequence of RV retrograde transsynaptic spread (Figure 2A).

### 3.3 Identification of long‐range input regions to NEDA of the ARH of male and female mice

**Figure 3:**
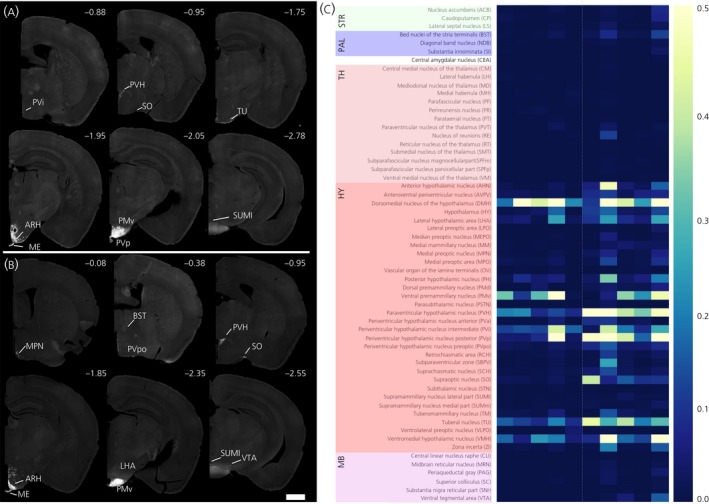
Whole‐brain monosynaptic inputs to neuroendocrine dopaminergic (NEDA) neurones in female and male mice. Representative coronal hemi‐sections of a female mouse brain (A), and a male mouse brain (B) depicting rabies green fluorescent protein (GFP) signal. Numberd on the top left indicate the distance from Bregma in mm. Scale bar = 1 mm. C, Heat‐map representation of the normalised counts of GFP‐positive cells. The counts were normalised by dividing the number of cells observed in a given region by the number of cells in the arcuate nucleus of the hypothalamus (ARH). The ARH was subsequently removed from analysis. The animals are arranged by sex. The first five animals are females in decreasing order of total number of cells detected and the last five animals are males in increasing order of total number of cells detected (left to right). The vast majority of monosynaptic inputs to NEDA neurones originate in the hypothalamus

All of our accepted injection sites were located adjacent to the dmARH (representative example in Figure 3A). There was no significant difference between the total numbers of normalised monosynaptic inputs detected in females vs males (*U* = 4, two‐tailed Mann‐Whitney *U* test). However, there was variability in some input regions within sex (Figure 3B). This was likely a result of experimental variation and different degrees of contamination by nearby regions containing Dat‐positive neurones. Therefore, in our analysis, we only included brain regions where we observed GFP‐labelled neurones in at least three animals (Figure 3C).

**Figure 4:**
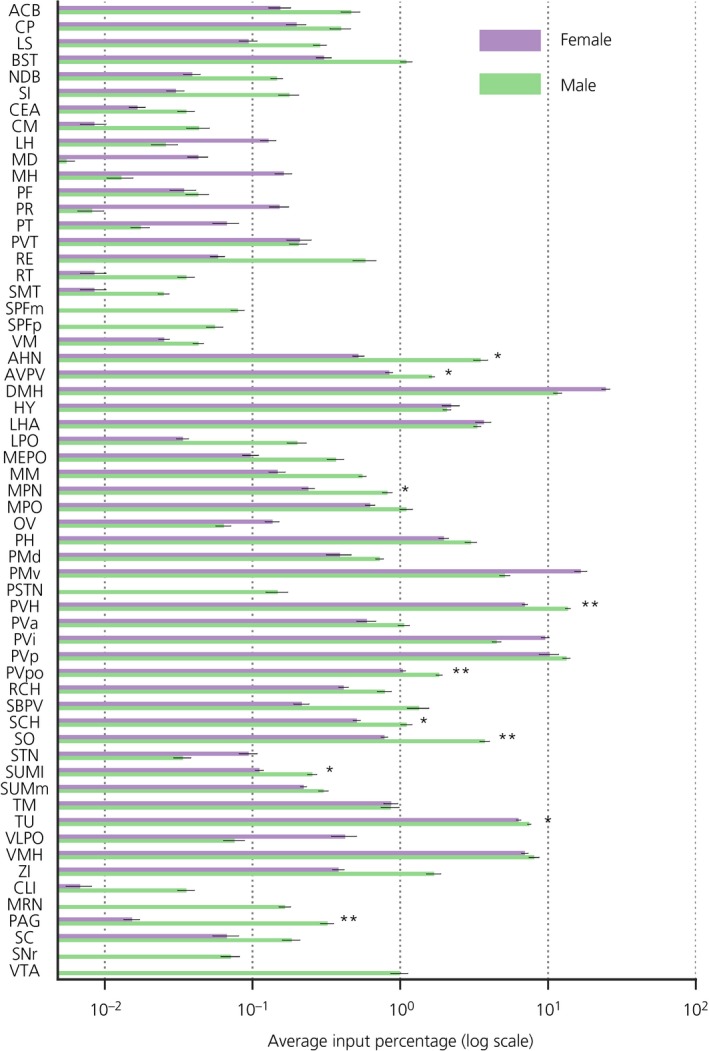
Monosynaptic inputs to neuroendocrine dopaminergic (NEDA) neurones are sexually dimorphic. Average input distribution of green fluorescent protein (GFP)‐positive cells by sex. Each brain region was averaged across sex and converted to a percentage value of total inputs by sex. To detect significant differences between males and females in each brain region, we performed a two‐tailed Mann‐Whitney *U* test and accepted significance when *U* ≤ 2 (*α = 0.05) and U = 0 (**α = 0.01). Ten regions were found to significantly contribute more inputs in males than in females: the anterior hypothalamic nucleus, the anteroventral periventricular nucleus, the medial preoptic nucleus, the paraventricular hypothalamic nucleus, the posterior periventricular nucleus, the supraoptic nucleus, the suprachiasmatic nucleus, the lateral supramammillary nucleus, the tuberal nucleus and the periaqueductal grey

We identified 59 regions reliably contributing inputs to the ARH. The cell counts are reported as average percentage input contribution (Figure 4). Average input contributions were calculated by first normalising cell counts by the ARH cell count and then the normalised values were averaged across animals. The percentage for each region is calculated by dividing the average of a given region by the sum of means of all regions. The regions contributing the greatest proportion of inputs in females were the dorsomedial hypothalamic nucleus (DMH) (24.7%), the ventral premammillary nucleus (PMv) (16.8%) and the periventricular hypothalamic nucleus posterior (PVp) (10.3%), whereas, in males, they were the PVH (13.7%), the PVp (13.3%) and the DMH (11.6%). In both male and female mice, presynaptic GFP‐positive neurones were mainly detected in the hypothalamus (AHN, ARH, AVPV, DMH, LPO, LHA, MEPO, MM, MPN, MPO, OV, PH, PMd, PMv, PVa, PVH, PVi, PVp, PVpo, PSTN, RCH, SBPV, SCH, SO, STN, SUMl, SUMm, TM, TU, VLPO, VMH, ZI) (for abbreviations, see Figure 3). In addition, approximately 2% of total inputs in both males and females originated from unnamed hypothalamic regions (HY). The striatum showed considerable inputs (ACB, CP, LS), as did the pallidum (BST, NDB and SI). The only amygdalar region that consistently contributed inputs was the central amygdala, whereas several regions of the thalamus contributed monosynaptic inputs (CM, LH, MD, MH, PF, PR, PT, PVT, RE, RT, SMT, VM). From the midbrain, the input regions detected in animals of both sexes were the superior colliculus (SC) and the ventrolateral PAG. Notably, no GFP‐positive cells were reliably detected in the olfactory bulb or the cerebellum.

### 3.4 Monosynaptic input patterns to NEDA are sexually dimorphic

To investigate the possibility that the distribution of monosynaptic inputs observed for male and female *dat‐cre* mice is sexually dimorphic, we performed a Mann‐Whitney *U* test on each region using normalised cell counts. Taking into consideration the size of our samples, we only accepted a significant difference where the *U* statistic is smaller than or equal to 2 (α = 0.05). According to this analysis, the following regions provided a greater share of inputs in males than in females: AHN (U = 1, *P* = 0.01), AVPV (U = 2, *P* = 0.018), MPN (U = 2, *P* = 0.018), PVH (U = 0, *P* = 0.006), PVpo (U = 0, *P* = 0.006), SO (U = 0, *P* = 0.006), SCH (U = 2, *P* = 0.018), SUMl (U = 2, *P* = 0.018), TU (U = 1, *P* = 0.01) and PAG (U = 0, *P* = 0.006) (Figure 4).

In addition, some regions (SPFp, SPFm, PSTN, VTA and MRN) were only detected in males, although one out of the five males did not display GFP‐positive cells in these regions. The substantia nigra reticular part was also only detected in males but, in this case, in three out of the five males. Given the sample size and choice of statistical test, these regions were not statistically different between males and females, even though the difference is striking. Furthermore, regions where the female contribution substantially exceeded on average the contribution in the male (MH, MD, PR, LH, PVi, PMv and DMH) were not found to be statistically significant.

## 4 DISCUSSION

The present study reports for the first time whole‐brain monosynaptic inputs to the NEDA of the ARH that control the release of the pituitary hormone PRL.

We chose to deploy the rabies monosynaptic tracing system in transgenic mice expressing Cre recombinase under the control of the Dat promoter to genetically define the NEDA population. Even though Dat‐positive neurones represent a subpopulation of ARH dopaminergic neurones,^37^ Dat activity is necessary to ensure proper physiological PRL function.^7^ The choice of Dat as a marker for NEDA renders our results highly relevant for studies concerning PRL regulation.

We reveal that, despite having a monomorphic number and density of NEDA neurones, the monosynaptic inputs to this population are sexually dimorphic in mice, opening up new avenues of research for understanding the role of PRL in the control of sex‐specific behaviours.

### 4.1 Distribution of NEDA neurones in mice is monomorphic

Recently, much effort has been dedicated to unravelling the neuronal circuitry underlying sexual dimorphic behaviours, as a result of the importance of these for the reproductive success of individuals and survival of the progeny. Dimorphisms in behaviour have been mostly associated with quantitative differences in cell numbers.^39^ By contrast, here, we have shown that the number and distribution of Dat‐positive NEDA neurones in mice is similar between males and females, in accordance with previous studies,^37^ implying that the dimorphism in PRL physiology, such as higher PRL concentration in the blood of females,^5^ and associated behaviours must arise from other sources. Still, it is likely that the output of mouse NEDA neurones is sexually dimorphic: in the rat, for example, female NEDA neurones produce higher levels of dopamine than their male counterparts.^34^

### 4.2 Identification of long‐range input regions to NEDA of the ARH of male and female mice

The ARH is an extensively studied area of the brain in diverse contexts and has a highly diverse cell population.^40^ In our study, the ARH had consistently high numbers of GFP‐positive only (non‐starter) cells, thus attesting to the high degree of intrinsic connectivity within the ARH.^29^ We focused our analysis on inputs originating outside the ARH because the RV system employed here is not adequate to perform local circuitry input mapping. To perfoem a local circuitry study, a different version of the RV system that employs a mutant TVA with reduced transfection efficiency is recommended^41^ to allow labelling of more sparse local inputs.

In female mice, the area contributing the most inputs to the ARH is the DMH, whereas, in males, it is the PVH. In both sexes, both of these areas contribute a substantial amount of inputs to NEDA neurones. Neuronal activity in the DMH and the PVH has been implicated in lactation.^42^ We propose that concomitant activation of these brain regions might result in direct signalling onto NEDA neurones and thus affect PRL release; the nature of the neurotransmitter released by the DMH and PVH remains to be clarified. Regarding males, the role of these brain areas in the context of the regulation of NEDA neurones is currently a mystery.

In rodents, dimorphisms have been identified in the number of neurones within brain regions or their projections that are relevant for dimorphic social behaviours.^39^ Indeed, in our study, we detected significant sexual dimorphisms in four of the six areas classically defined as the mammalian social brain network^22^: the MPN (representing medial preoptic regions); the AHN (representing the anterior hypothalamus); the PVpo (representing the ventromedial hypothalamus); and the PAG (representing the midbrain). The missing nodes are the lateral septal nucleus (LS) and the bed nuclei of the stria terminalis (BST). In our study, we observed that both the LS and the BST have a larger number of cells projecting to NEDA neurones in males, although we are not able to statistically support this dimorphism. However, our results regarding the BST are in agreement with reports stating a sexual dimorphic nature of BST projections to the dorsomedial and ventrolateral ARH^43^ in rats. Unequivocally, we now show that at least some of the BST‐to‐ARH projecting neurones synapse onto NEDA neurones in mice.

The MPN participates in sexual behaviour in both males and females.^44^ Because PRL is released during sexual behaviour,^45^ it is not surprising that MPN neurones synapse directly onto NEDA neurones. The AHN is a sexually dimorphic nucleus^46^ with reported sparse projections to the ARH in male rats.^47^ We find evidence supporting the existence of these projections in both male and female mice and further specify that part of these projections terminate in NEDA neurones. The periventricular areas (PVa, PVp and PVpo) together with the SO, the TU and the SUMl are themselves part of the neuroendocrine system.^7^

Given the diverse neurochemical production profiles of the aforementioned areas (TRH, somatostatin, GnRH, leptin, kisspeptin, oxytocin and NPY among others), the role of their inputs into NEDA neurones of the ARH probably hinges on understanding how the neuroendocrine system coordinates multiple hormonal systems. The relationship between the brain social network and the NEDA neurones is likely a multifaceted one. NEDA neurones are positioned to not only sense the global physiologic state of the animal (possibly via contact with the cerebrospinal fluid and the blood through the ME), but also enact a brainwide response by neuroendocrine action through PRL. Furthermore, in females, several of the regions projecting to NEDA neurones identified in the present study are also PRL‐responsive.^21^ This suggests the existence of a feedback mechanism in the brain influencing the control of PRL release by PRL‐responsive regions that contact NEDA neurones, in addition to direct PRL action onto NEDA neurones.

### 4.3 The role of PRL in male sexual behaviour

Even though PRL is paramount for the regulation of sexual behaviour and sex‐specific behaviours, little is known about the role of this hormone in male physiology. Curiously, we detected inputs that consistently appeared in males but not in females. One of these input areas, the suprafascicular nucleus (SPF), has been reported as having direct projections to dynorphin‐positive neurones of the ARH in non‐lactating females^48^ but not dopaminergic neurones. Because the SPF has been implicated in the control of ejaculation,^44^ it is tempting to speculate that the projection from the SPF onto NEDA neurones controls the release of PRL during copulation. PRL release during sexual behaviour could be involved in the priming of the male brain for paternity, as appears to be the case in females.^15^ This view is in accordance with the observation that sexually experienced males have decreased rates of infanticide.^49^

### 4.4 Caveats of the monosynaptic rabies tracing technique and further work

Despite the specificity and potential of the rabies monosynaptic tracing system,^31^ there are some limitations to this methodology, in particular, the completeness of input coverage to a given population of starter cells, as well as in the types of synapses the RV can traverse.^32^ Therefore, we do not claim to have uncovered all the brain regions synapsing onto Dat‐positive NEDA neurones, but rather that the regions we have uncovered are reliable monosynaptic inputs outside the ARH.

The main challenge of the present study is the heterogeneity observed within animals of the same sex. We attribute this variation to the inherent technical challenges in the methods used. Although we set our injections to deliver the viruses on the dorso‐medial regions of the ARH, slight rostrocaudal variations as a result of experimental bias might have occurred. This resulted in different levels of contamination by nearby Dat‐positive nuclei observed in different animals. To obviate this pitfall, the use of intersectional genetic strategies would be ideal. This would help to distinguish not only between GABA expressing from non‐GABA expressing NEDA neurones,^50^ but also exclude PMv Dat‐positive neurone contamination, because this nucleus does not display GABA vesicular transporter expression.^20^ Another possible source of confounding results could be the inadvertent retrograde transfection of Dat‐positive fibres of passage, which might have happened to a different extent across experiments. For example, we detected mCherry signal in GFP‐negative populations of the ventral tegmental area in some of our animals, suggesting that Dat‐positive terminals can be infected with the AAV carrying the TVA transgene and could in principle serve as a starter population outside the ARH. To mitigate this issue, we only considered brain regions where we observed GFP‐positive neurones in at least three of the animals.

In addition, the choice of Dat as marker might result in incomplete coverage of all dopaminergic neurones participating in PRL regulation. For example, a brain area reported in female rats that synapses onto tyrosine hydroxylase‐positive neurones of the ARH is the intergeniculate leaflet of the lateral geniculate,^38^ an area related to circadian regulation. However, in our study, we did not reliably find GFP‐positive neurones in this area in females, only in two male animals (see Supporting information, Table S1). It is possible that this particular discrepancy is as a result of the fact that not all of the ARH Th‐positive neurones are Dat‐positive.^37^

Several experiments can be performed as a follow‐up to ensure that the regions reported as monosynaptic inputs are indeed synapsing onto NEDA neurones, starting with the injection of anterograde traveling viral particles in the brain regions identified in this study.

## 5 CONCLUSIONS

The present study has laid the necessary grounds for the future detailed investigation of PRL function in a broad variety of contexts, ranging from sexual behaviour to maternal care and drug development efforts adequate to both male and female physiology. Importantly, as far as we know, this is the first instance where the rabies monosynaptic tracing system was used to unveil the sexual dimorphism of synaptic inputs into a neuronal population, emphasising the importance of considering sex as a variable in studies of neuronal connectivity. Finally, despite the clear existence of dimorphisms in behaviour, there are very few cases where the underlying circuitry has been identified and examples of dimorphisms in connectivity onto a monomorphic population are rare. Therefore, besides the contribution to our knowledge regarding the regulation of PRL physiology and associated in behaviours, the present study also puts forward an interesting candidate circuit for interrogating basic questions related to sex‐specific development and wiring of neuronal circuits.

## CONFLICT OF INTERESTS

The authors declare that they have no conflicts of interest.
