# Supplementary material for: Sexually dimorphic neuronal inputs to the neuroendocrine dopaminergic system governing prolactin release
Source: J Neuroendocrinol. 2019 Sep 2;31(10):e12781. doi: 10.1111/jne.12781 (PMC6851580; doi:10.1111/jne.12781)
Supplement: Supplementary file 1 [file JNE-31-na-s001.docx]

**Supplementary Information**

| Region | F1 | F2 | F3 | F4 | F5 | M1 | M2 | M3 | M4 | M5 |
| --- | --- | --- | --- | --- | --- | --- | --- | --- | --- | --- |
| AAA | 0 | 0 | 0 | 0 | 0 | 0 | 0 | 0 | 3 | 0 |
| ACAd5 | 0 | 0 | 0 | 0 | 0 | 0 | 0 | 0 | 1 | 0 |
| ACB | 0 | 3 | 0 | 0 | 2 | 0 | 0 | 5 | 78 | 7 |
| AD | 0 | 0 | 0 | 0 | 0 | 0 | 0 | 0 | 1 | 0 |
| AHN | 4 | 23 | 3 | 1 | 4 | 25 | 44 | 61 | 161 | 170 |
| AM | 0 | 0 | 0 | 0 | 0 | 0 | 0 | 0 | 0 | 1 |
| AON | 0 | 1 | 0 | 0 | 0 | 0 | 0 | 0 | 0 | 0 |
| APN | 0 | 0 | 0 | 0 | 0 | 0 | 0 | 0 | 2 | 1 |
| ARH | 1143 | 1429 | 981 | 282 | 145 | 416 | 2208 | 1306 | 990 | 350 |
| AUD | 0 | 0 | 0 | 0 | 0 | 0 | 0 | 0 | 1 | 0 |
| AVP | 0 | 0 | 0 | 0 | 0 | 0 | 0 | 0 | 0 | 6 |
| AVPV | 33 | 34 | 13 | 0 | 3 | 36 | 143 | 28 | 74 | 40 |
| Aid | 0 | 0 | 0 | 0 | 0 | 0 | 0 | 0 | 1 | 0 |
| BA | 0 | 0 | 0 | 0 | 0 | 0 | 0 | 0 | 5 | 3 |
| BST | 2 | 7 | 4 | 0 | 3 | 8 | 24 | 17 | 119 | 28 |
| CA3 | 0 | 0 | 0 | 0 | 0 | 0 | 0 | 0 | 1 | 0 |
| CEA | 0 | 1 | 1 | 0 | 0 | 0 | 0 | 0 | 5 | 1 |
| CLI | 0 | 1 | 0 | 0 | 0 | 0 | 0 | 0 | 5 | 1 |
| CM | 1 | 0 | 0 | 0 | 0 | 0 | 0 | 0 | 1 | 3 |
| CP | 5 | 23 | 0 | 0 | 0 | 0 | 0 | 6 | 71 | 4 |
| CSm | 0 | 0 | 0 | 0 | 0 | 0 | 0 | 0 | 7 | 0 |
| CUN | 0 | 0 | 0 | 0 | 0 | 0 | 0 | 0 | 0 | 1 |
| DG | 0 | 0 | 0 | 0 | 0 | 0 | 0 | 0 | 5 | 0 |
| DMH | 692 | 260 | 430 | 62 | 158 | 43 | 550 | 573 | 877 | 310 |
| DR | 0 | 0 | 0 | 0 | 0 | 0 | 0 | 2 | 8 | 0 |
| EW | 0 | 0 | 0 | 0 | 0 | 0 | 0 | 0 | 1 | 0 |
| FN | 0 | 0 | 0 | 0 | 0 | 0 | 0 | 0 | 1 | 0 |
| FS | 0 | 1 | 0 | 0 | 0 | 0 | 0 | 0 | 0 | 0 |
| GPe | 0 | 1 | 0 | 0 | 0 | 0 | 0 | 0 | 3 | 0 |
| GPi | 0 | 0 | 0 | 0 | 0 | 1 | 0 | 0 | 0 | 0 |
| GRN | 0 | 0 | 0 | 0 | 0 | 0 | 0 | 0 | 1 | 1 |
| HY | 21 | 12 | 35 | 0 | 24 | 47 | 47 | 41 | 126 | 58 |
| IF | 0 | 0 | 0 | 0 | 0 | 0 | 0 | 0 | 6 | 0 |
| IGL | 0 | 0 | 0 | 0 | 0 | 0 | 0 | 0 | 4 | 1 |
| IMD | 2 | 0 | 0 | 0 | 0 | 0 | 0 | 0 | 0 | 0 |
| IPN | 0 | 1 | 0 | 0 | 0 | 0 | 0 | 0 | 4 | 0 |
| LGv | 0 | 0 | 0 | 0 | 0 | 0 | 0 | 0 | 3 | 1 |
| LH | 2 | 12 | 3 | 0 | 0 | 0 | 0 | 0 | 0 | 2 |
| LHA | 58 | 55 | 9 | 7 | 37 | 40 | 115 | 113 | 255 | 85 |
| LPO | 2 | 1 | 1 | 0 | 0 | 0 | 0 | 2 | 34 | 3 |
| LS | 0 | 4 | 0 | 0 | 1 | 2 | 3 | 2 | 24 | 11 |
| MA | 0 | 0 | 0 | 0 | 0 | 0 | 0 | 0 | 2 | 0 |
| MARN | 0 | 0 | 0 | 0 | 0 | 0 | 2 | 0 | 0 | 2 |
| MD | 1 | 0 | 0 | 1 | 0 | 0 | 1 | 1 | 0 | 0 |
| MEAav | 0 | 0 | 0 | 0 | 0 | 0 | 1 | 0 | 0 | 0 |
| MEPO | 2 | 2 | 0 | 0 | 1 | 1 | 2 | 6 | 16 | 20 |
| MH | 7 | 0 | 0 | 3 | 0 | 0 | 0 | 0 | 0 | 1 |
| MM | 0 | 13 | 6 | 0 | 0 | 0 | 57 | 55 | 26 | 10 |
| MOs | 0 | 0 | 0 | 0 | 0 | 0 | 0 | 0 | 3 | 0 |
| MPN | 4 | 9 | 1 | 0 | 2 | 10 | 24 | 9 | 67 | 25 |
| MPO | 10 | 27 | 9 | 0 | 4 | 4 | 25 | 9 | 89 | 44 |
| MRN | 0 | 0 | 0 | 0 | 0 | 2 | 0 | 2 | 13 | 6 |
| MS | 0 | 0 | 0 | 0 | 0 | 0 | 0 | 0 | 2 | 3 |
| MeAad | 0 | 0 | 0 | 0 | 0 | 0 | 0 | 0 | 5 | 2 |
| NB | 0 | 0 | 0 | 0 | 0 | 0 | 0 | 0 | 1 | 0 |
| NDB | 3 | 2 | 0 | 0 | 0 | 2 | 2 | 0 | 15 | 4 |
| NI | 0 | 0 | 0 | 0 | 0 | 0 | 0 | 0 | 1 | 0 |
| NLL | 0 | 0 | 0 | 0 | 0 | 0 | 0 | 0 | 1 | 0 |
| NOT | 0 | 0 | 0 | 0 | 0 | 0 | 0 | 0 | 0 | 2 |
| ORB | 0 | 0 | 0 | 0 | 3 | 0 | 0 | 0 | 3 | 0 |
| OT | 0 | 0 | 0 | 0 | 0 | 1 | 0 | 0 | 15 | 0 |
| OV | 7 | 0 | 1 | 0 | 1 | 0 | 20 | 4 | 2 | 0 |
| PAA | 0 | 0 | 0 | 0 | 0 | 0 | 1 | 0 | 2 | 0 |
| PAG | 1 | 1 | 0 | 0 | 0 | 2 | 2 | 4 | 39 | 8 |
| PB | 0 | 0 | 0 | 0 | 0 | 3 | 0 | 0 | 0 | 0 |
| PCG | 0 | 0 | 0 | 0 | 0 | 0 | 0 | 0 | 0 | 1 |
| PF | 0 | 0 | 0 | 1 | 0 | 0 | 2 | 0 | 0 | 3 |
| PH | 97 | 19 | 42 | 0 | 9 | 13 | 133 | 41 | 198 | 120 |
| PIR | 0 | 0 | 0 | 0 | 0 | 0 | 0 | 0 | 2 | 0 |
| PMd | 0 | 2 | 38 | 0 | 0 | 8 | 30 | 26 | 67 | 14 |
| PMv | 122 | 475 | 308 | 3 | 139 | 0 | 514 | 468 | 473 | 21 |
| PO | 0 | 0 | 0 | 0 | 0 | 0 | 0 | 0 | 2 | 0 |
| PP | 0 | 0 | 0 | 0 | 0 | 0 | 0 | 0 | 1 | 0 |
| PPN | 0 | 0 | 0 | 0 | 0 | 0 | 0 | 0 | 1 | 2 |
| PPY | 0 | 0 | 0 | 0 | 0 | 0 | 0 | 0 | 1 | 0 |
| PR | 4 | 0 | 12 | 0 | 0 | 0 | 4 | 0 | 0 | 0 |
| PRN | 0 | 0 | 0 | 0 | 0 | 0 | 0 | 0 | 5 | 1 |
| PSTN | 0 | 0 | 0 | 0 | 0 | 1 | 0 | 1 | 1 | 10 |
| PT | 0 | 0 | 0 | 0 | 1 | 0 | 0 | 0 | 1 | 1 |
| PVH | 248 | 292 | 82 | 23 | 19 | 394 | 757 | 528 | 499 | 286 |
| PVT | 1 | 0 | 0 | 0 | 3 | 0 | 2 | 0 | 13 | 11 |
| PVa | 4 | 6 | 5 | 0 | 7 | 37 | 57 | 20 | 1 | 36 |
| PVi | 164 | 167 | 124 | 47 | 63 | 10 | 189 | 255 | 267 | 147 |
| PVp | 51 | 66 | 83 | 9 | 123 | 211 | 370 | 565 | 600 | 430 |
| PVpo | 19 | 37 | 9 | 6 | 5 | 33 | 100 | 55 | 83 | 54 |
| RCH | 3 | 8 | 10 | 1 | 3 | 3 | 21 | 22 | 31 | 38 |
| RE | 2 | 1 | 0 | 1 | 0 | 0 | 14 | 0 | 5 | 41 |
| RL | 0 | 0 | 0 | 0 | 0 | 0 | 0 | 1 | 0 | 3 |
| RM | 0 | 0 | 0 | 0 | 0 | 0 | 0 | 0 | 2 | 8 |
| RO | 0 | 0 | 0 | 0 | 0 | 0 | 0 | 0 | 1 | 0 |
| RSP | 0 | 0 | 0 | 0 | 0 | 0 | 0 | 0 | 0 | 2 |
| RT | 1 | 0 | 0 | 0 | 0 | 0 | 0 | 0 | 5 | 1 |
| SBPV | 8 | 2 | 0 | 0 | 2 | 3 | 4 | 24 | 11 | 90 |
| SC | 0 | 0 | 0 | 0 | 1 | 0 | 0 | 0 | 12 | 10 |
| SCH | 3 | 13 | 9 | 3 | 3 | 18 | 23 | 31 | 32 | 47 |
| SGN | 0 | 0 | 0 | 0 | 0 | 0 | 0 | 0 | 2 | 0 |
| SI | 0 | 3 | 1 | 0 | 0 | 0 | 1 | 1 | 32 | 2 |
| SMT | 1 | 0 | 0 | 0 | 0 | 0 | 2 | 1 | 1 | 1 |
| SNc | 0 | 0 | 0 | 0 | 0 | 1 | 0 | 0 | 5 | 0 |
| SNr | 0 | 0 | 0 | 0 | 0 | 0 | 3 | 3 | 12 | 0 |
| SO | 21 | 31 | 9 | 1 | 4 | 171 | 201 | 197 | 100 | 25 |
| SPA | 1 | 0 | 0 | 0 | 0 | 0 | 0 | 0 | 0 | 5 |
| SPFm | 0 | 0 | 0 | 0 | 0 | 0 | 16 | 1 | 1 | 3 |
| SPFp | 0 | 0 | 0 | 0 | 0 | 0 | 1 | 3 | 1 | 3 |
| STN | 0 | 4 | 0 | 0 | 1 | 1 | 0 | 0 | 5 | 0 |
| SUB | 0 | 0 | 0 | 0 | 0 | 0 | 0 | 0 | 4 | 5 |
| SUMl | 2 | 3 | 4 | 1 | 0 | 4 | 17 | 35 | 9 | 1 |
| SUMm | 2 | 5 | 7 | 1 | 1 | 6 | 20 | 7 | 32 | 2 |
| TM | 15 | 20 | 7 | 0 | 8 | 0 | 34 | 42 | 0 | 50 |
| TT | 0 | 0 | 0 | 0 | 0 | 0 | 0 | 0 | 4 | 0 |
| TU | 133 | 225 | 80 | 25 | 30 | 194 | 439 | 514 | 302 | 100 |
| VISC | 0 | 0 | 0 | 0 | 0 | 0 | 0 | 0 | 1 | 0 |
| VLPO | 0 | 0 | 1 | 12 | 0 | 0 | 1 | 0 | 2 | 5 |
| VM | 1 | 1 | 1 | 0 | 0 | 0 | 8 | 0 | 3 | 1 |
| VMH | 69 | 239 | 232 | 15 | 29 | 35 | 270 | 224 | 608 | 276 |
| VTA | 0 | 0 | 0 | 0 | 0 | 0 | 8 | 66 | 155 | 3 |
| ZI | 12 | 3 | 6 | 0 | 3 | 3 | 12 | 10 | 149 | 71 |
| Total | 2984 | 3545 | 2567 | 505 | 842 | 1789 | 6524 | 5387 | 6922 | 3145 |

**Table S1- Total GFP-Positive cell counts in Rabies Injected Animals** Absolute counts of GFP-positive cells by brain region for female (F1-F5) and male (M1-M5) Rabies Virus injected animals used in the present study. Abbreviations follow the nomenclature of the Allen Brain Atlas^1^.

**Abbreviations:**

| AAA - Anterior amygdalar area   \| ACAd5 - Anterior cingulate area, dorsal part, layer 5 \| \| --- \| |
| --- | --- |
| ACB - Nucleus accumbens |
| AD - Anterodorsal nucleus |
| AHN - Anterior hypothalamic nucleus |
| Aid - Agranular insular area, dorsal part |
| AON - Anterior olfactory nucleus |
| APN - Anterior pretectal nucleus |
| ARH - Arcuate hypothalamic nucleus |
| AUD - Auditory areas |
| AVPV - Anteroventral periventricular nucleus |
| BA - Bed nucleus of the accessory olfactory tract |
| BST - Bed nuclei of the stria terminalis |
| CA3 - Field CA3 |
| CEA - Central amygdalar nucleus |
| CLI - Central linear nucleus raphe |
| CM - Central medial nucleus of the thalamus |
| CP - Caudoputamen |
| CSm - Superior central nucleus raphe, medial part |
| DG - Dentate gyrus |
| DMH - Dorsomedial nucleus of the hypothalamus |
| DR - Dorsal nucleus raphe |
| EW - Edinger-Westphal nucleus |
| FN - Fastigial nucleus |
| FS - Fundus of Striatum |
| GPe - Globus pallidus external segment |
| GPi - Globus pallidus internal segment |
| GRN - Gigantocellular reticular nucleus |
| HY - Hypothalamus |
| IF - Interfascicular nucleus raphe |
| IGL - Intergeniculate leaflet of the lateral geniculate complex |
| IMD - Intermediodorsal nucleus of the thalamus |
| IPN - Interpeduncular nucleus |
| LGv - Ventral part of the lateral geniculate complex |
| LH - Lateral habenula |
| LHA - Lateral hypothalamic area |
| LPO - Lateral preoptic area |
| LS - Lateral septal nucleus |
| MA - Magnocellular nucleus |
| MARN - Magnocellular reticular nucleus |
| MD - Mediodorsal nucleus of thalamus |
| MEAad - Medial amygdalar nucleus, anterodorsal part |
| MEAav - Medial amygdalar nucleus anteroventral part |
| MEPO - Median preoptic nucleus |
| MH - Medial habenula |
| MM - Medial mammillary nucleus |
| MOs - Secondary motor area |
| MPN - Medial preoptic nucleus |
| MPO - Medial preoptic area |
| MRN - Midbrain reticular nucleus |
| MS - Medial septal nucleus |
| NB - Nucleus of the brachium of the inferior colliculus |
| NDB - Diagonal band nucleus |
| NI - Nucleus incertus |
| NLL - Nucleus of the lateral lemniscus |
| NOT - Nucleus of the optic tract |
| ORB - Orbital area |
| OT - Olfactory tubercle |
| OV - Vascular organ of the lamina terminalis |
| PAA - Piriform amygdalar area |
| PAG - Periaqueductal gray |
| PB - Parabrachial nucleus |
| PCG - Pontine central gray |
| PF - Parafascicular nucleus |
| PH - Posterior hypothalamic nucleus |
| PIR - Piriform area |
| PMd - Dorsal premammillary nucleus |
| PMv - Ventral premammillary nucleus |
| PO - Posterior complex of the thalamus |
| PP - Peripeduncular nucleus |
| PPN - Pedunculopontine nucleus |
| PPY - Parapyramidal nucleus |
| PR - Perireunensis nucleus |
| PRN - Pontine reticular nucleus |
| PSTN - Parasubthalamic nucleus |
| PT - Parataenial nucleus |
| PVa - Periventricular hypothalamic nucleus anterior |
| PVH - Paraventricular hypothalamic nucleus |
| PVi - Periventricular hypothalamic nucleus intermediate |
| PVp - Periventricular hypothalamic nucleus posterior |
| PVpo - Periventricular hypothalamic nucleus preoptic |
| PVT - Paraventricular nucleus of the thalamus |
| RCH - Retrochiasmatic area |
| RE - Nucleus of reunions |
| RL - Rostral linear nucleus raphe |
| RM - Nucleus raphe magnus |
| RO - Nucleus raphe obscurus |
| RT - Reticular nucleus of the thalamus |
| SBPV - Subparaventricular zone |
| SC - Superior colliculus |
| SCH - Suprachiasmatic nucleus |
| SGN - Suprageniculate nucleus |
| SI - Substantia innominata |
| SMT - Submedial nucleus of the thalamus |
| SNc - Substantia nigra compact part |
| SNr - Substantia nigra reticular part |
| SO - Supraoptic nucleus |
| SPA - Subparafascicular area |
| SPFm - Subparafascicular nucleus magnocellularpart |
| SPFp - Subparafascicular nucleus parvicellular part |
| STN - Subthalamic nucleus |
| SUB - Subiculum |
| SUMl - Supramammillary nucleus lateral part |
| SUMm - Supramammillary nucleus medial part |
| TM - Tuberomammillary nucleus |
| TT - Taenia tecta |
| TU - Tuberal nucleus |
| VISC - Visceral area |
| VLPO - Ventrolateral preoptic nucleus |
| VM - Ventral medial nucleus of the thalamus |
| VMH - Ventromedial hypothalamic nucleus |
| VTA - Ventral tegmental area |
| ZI - Zona incerta |
|  |
|  |
|  |

**Reference**

**1.** Lein ES, Hawrylycz MJ, Ao N, et al. Genome-wide atlas of gene expression in the adult mouse brain. *Nature.* 2006,;445:168-176.
